# Second Primary Malignancies in Patients with Pancreatic Neuroendocrine Neoplasms: A Population-Based Study on Occurrence, Risk Factors, and Prognosis

**DOI:** 10.1155/2021/1565089

**Published:** 2021-10-31

**Authors:** Tulan Hu, Wei Wang, Chiyi He

**Affiliations:** Department of Gastroenterology, Yijishan Hospital of Wannan Medical College, Wuhu 241000, China

## Abstract

**Background:**

This study aimed to evaluate the risk factors of developing second primary malignancies (SPMs) among patients with pancreatic neuroendocrine neoplasms (pNENs) and the prognosis of pNENs patients with SPMs (pSPMs) using data from the Surveillance, Epidemiology, and End Results (SEER) database.

**Methods:**

Data from patients diagnosed with pNENs between 1988 and 2016 were extracted. A case-control study was conducted to investigate the risk factors of developing SPMs among patients with pNENs. Meanwhile, cox regression analysis was also conducted to obtain the independent prognostic factors in pSPMs.

**Results:**

Of 7,630 patients with pNENs, 326 developed SPMs. Patients with pNENs who had not undergone surgery and had been diagnosed in recent periods had a higher risk of developing SPMs. The following independent prognostic predictors for pSPMs were identified: age, latency period, SEER stage, radiotherapy, and surgery.

**Conclusions:**

These findings may improve the surveillance of risk factors for developing SPMs in patients with pNENs and the prognostic risk factors in pSPMs.

## 1. Introduction

Pancreatic neuroendocrine neoplasms (pNENs) are rare tumors with an annual incidence of approximately 0.5 per 100,000 people [1, 2]. The incidence of pNENs continues to rise, which increased from 0.1 to 0.6 cases per 100,000 annually from 1994 to 2009 [3]. Because of clinical and pathological heterogeneity, choices of treatment should be personalized for patients with pNENs [4, 5].

Current treatment strategies include surgery and systemic therapies such as cytotoxic chemotherapy, targeted therapy, peptide receptor radionuclide therapy (PRRT), and somatostatin analogs (SSAs) [6, 7]. Surgery has been considered as the mainstay treatment modality for pNENs, especially for localized disease and is commonly recommended for localized nonfunctional pNENs with tumor size >2 cm and functional pNENs, while active surveillance is recommended for nonfunctional pNENs with tumor size <2 cm according to the European Neuroendocrine Tumor Society and the North American Neuroendocrine Tumor Society guidelines [8–12]. Chemotherapy and targeted therapy could inhibit specific molecular pathways to impede pNENs progression; PRRT is a novel treatment modality that binds to pNENs with overexpressed somatostatin receptor and then emits localized radiation [6, 13]; SSAs demonstrate antisecretory properties and antiproliferative potency, as a frontline treatment modality for advanced or metastatic pNENs; however, sequencing of treatment with chemotherapy, targeted therapy, and PRRT requires further investigation [6].

In the past decades, mortality for pNENs has decreased over time, reflecting improvement in therapies [1]. However, among pNENs survivors, one of the complications is developing second primary malignancies (SPMs) that the incidence has approximately doubled from 1975 to 2009 [14, 15]. The risk factors of developing SPMs in patients with pNENs and prognostic factors in pNENs patients with SPMs (pSPMs) have not been elucidated. In this study, we used data from the Surveillance, Epidemiology, and End Results (SEER) database to investigate the risk factors of developing SPMs in patients with pNENs and evaluate the independent prognostic predictors in pSPMs.

## 2. Materials and Methods

### 2.1. Study Population

**Figure 1:**
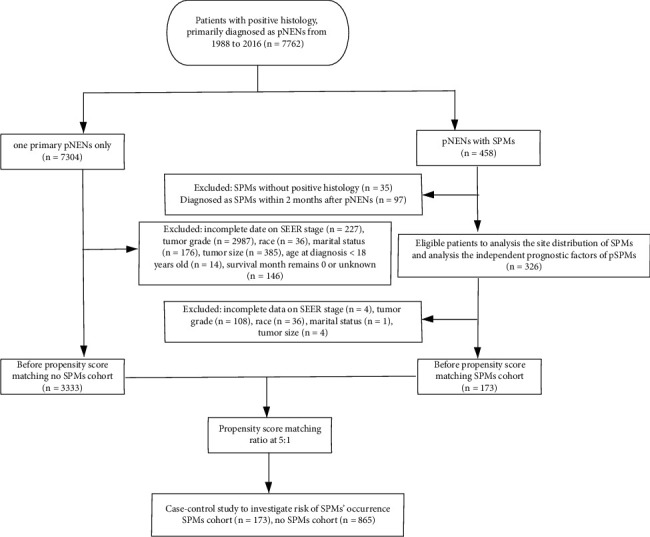
Flowchart of patients' inclusion and exclusion.

Patients diagnosed with pNEN as the first primary tumor between 1988 and 2016 were screened from the SEER database using the SEER *∗* Stat (8.3.8) software. The International Classification of Disease for Oncology, 3rd edition (ICD-O-3) codes C25.0–C25.9, was used to recognize the “pancreas.” Neuroendocrine neoplasms were identified using the following codes: 8012, 8013, 8041, 8150, 8156, and 8240–8249. The following variables were considered: year of diagnosis, sex, age at diagnosis, patient race, site of the primary tumor, surgery, chemotherapy, radiotherapy, marital status, SEER stage, tumor size, tumor grade, survival status, overall survival (OS) time, latency period, tumor function, and tumor type. OS was defined as the interval from diagnosis to death or last follow-up [16]. SPM was defined as SPM diagnosed after a latency period longer than 2 months after the diagnosis of the primary pNEN [17]. The procedures of inclusion and exclusion of patients are shown in Figure 1.

### 2.2. Statistical Analysis

Propensity-score matching was performed to control the confounding bias using “MatchIt” R package, and the risk factors for developing SPMs among patients with pNENs were evaluated in a case-control study. pNENs patients without SPMs (pwSPMs) and pSPMs were identified as controls and cases, respectively. Patients alive or dead were identified as censored at the end of the follow-up period. Time to event was defined as the interval time between the diagnosis of primary pNENs and the development of SPMs. Clinicopathological differences between the two groups were analyzed using Chi-square tests. Cox proportional hazards regression analyses were performed to investigate the risk factors of occurrence of SPMs among patients with pNENs after propensity-score matching using “survival” R package. Variables with *p* values <0.2 in the univariate analysis were exported to the multivariate analysis. Each variable was estimated by the hazard ratios (HRs) with corresponding 95% confidence intervals (CIs).

Next, cox proportional hazards analyses were conducted to estimate predictors for OS in pSPMs. The variables with a *p* value <0.2 in the univariate analysis were selected for the multivariate analysis. A nomogram was generated to predict 5-, 10-, and 15-year OS rates using independent prognostic predictors by “rms” R package [18–20]. The performance of the nomogram was evaluated by calibration plots, receiver operating characteristic (ROC) curves, and concordance index (C-index) using “survival” and “timeROC” R packages [21]. The C-index was adjusted for internal validation (bootstraps with 1000 resamples). In addition, decision curve analysis (DCA) was used to obtain the net benefit and net reduction using “rmda” R package [22]. Furthermore, the restricted mean survival time (RMST) was calculated to illustrate the survival difference between pSPMs and pwSPMs using “survRM2” R package [23]. Values with *p* < 0.05 were considered statistically significant unless otherwise stated. Statistical analyses were performed using R software (version 4.0.1).

## 3. Results

### 3.1. Patients' Characteristics

**Figure 2:**
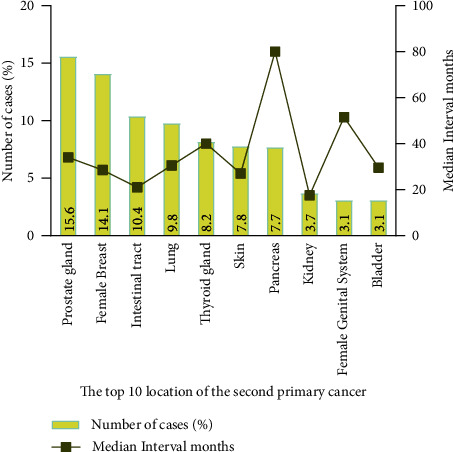
The site distribution and median interval time of second primary malignancies in patients with pancreatic neuroendocrine neoplasms.

A total of 7,630 patients were included, out of which 326 (4.27%) patients developed SPMs, The clinicopathological characteristics of pSPMs are presented in Table 1. Most of them were White (82.1%), married (72.39%), and had undergone surgery (72.7%). As shown in Figure 2, the most common SPM site was the prostate gland (15.5%), followed by the female breast (14.3%) and the intestinal tract (10.2%), and the median time intervals were 34, 28.5, and 21 months, respectively. Two-thirds of the SPMs occurred within 5 years after the diagnosis of pNENs. Compared with the longest median interval of 88 months for the SPM site in the pancreas, the SPM site in the kidney had the shortest median interval of 17.5 months for developing SPMs.

### 3.2. Risk Factors for Developing SPMs after pNENs Diagnosis

**Figure 3:**
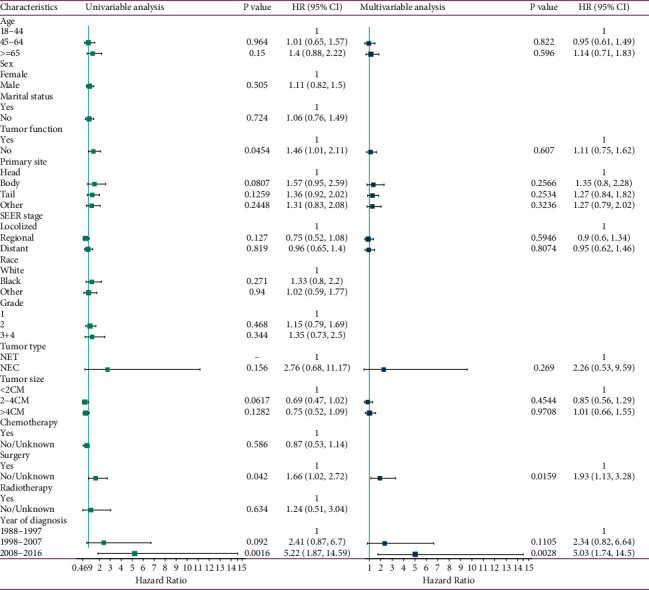
Univariable and multivariable cox proportional hazards analyses were performed to estimate risk factors of second primary malignancies development in pancreatic neuroendocrine neoplasms patients.

The matched group included a total of 1,038 patients, of which 173 and 865 patients had pSPMs and pwSPMs, respectively. Patients' characteristics are listed in Table 2. After matching, no statistical differences were observed between the clinicopathological parameters of the two cohorts. Results of cox regression analysis showed that treatment without surgery and diagnosed in recent periods were positively correlated with an increased risk of developing SPMs after pNEN diagnosis (Figure 3).

### 3.3. Restricted Mean Survival Time Curve

**Figure 4:**
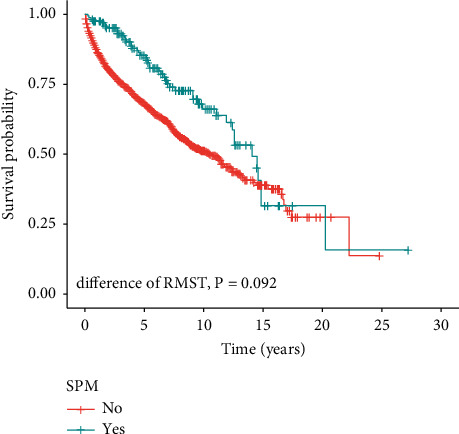
The survival curves of pSPMs and pwSPMs by restricted mean survival time. pSPMs: pNENs patients with second primary malignancies; pwSPMs: pNENs patients without second primary malignancies.

Survival curves were plotted to further compare the OS time between pSPMs and pwSPMs. As shown in Figure 4, pSPMs survive longer than pwSPMs with an RMST of 5-, 10-, 15-, and 20-year truncation times (all *p* < 0.01). However, within the maximum truncation time, a significant difference in the outcome was not observed between the two cohorts (*p*=0.092).

### 3.4. Independent Predictors for Prognosis

**Figure 5:**
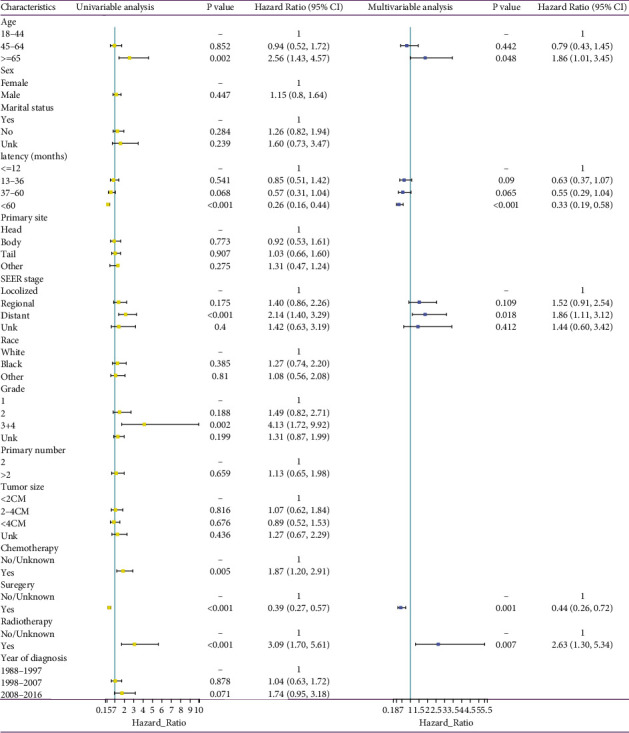
Univariable and multivariable cox proportional hazards analyses were performed to investigate independent prognostic predictors in pNENs patients with second primary malignancies.

As shown in Figure 5, parameters such as age, latency period, SEER stage, tumor grade, year of diagnosis, chemotherapy, radiotherapy, and surgery were related to the OS in the univariate cox analysis (*p* < 0.02). The abovementioned variables were further subjected to multivariate cox analysis, and the results showed that patients aged ≥65 years, with a distant SEER stage and who had received radiotherapy, were independently associated with an unfavorable prognosis; meanwhile, patients with a longer latency period and who had received surgery were independently associated with a better OS.

### 3.5. Development and Validation of the Prognostic Nomogram

**Figure 6:**
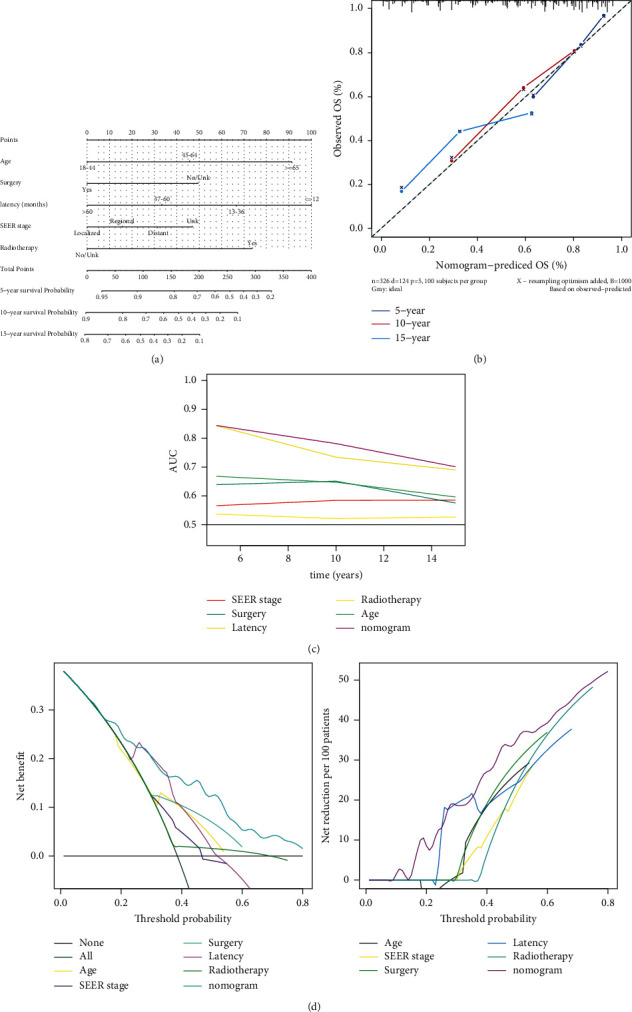
Construction and validation of the nomogram. (a) Nomogram predicting 5-, 10-, and 15-year OS rates in pSPMs. (b) Calibration plots. (c) ROC curves of the nomogram and clinicopathological factors. (d) Decision curves for net benefit and net reduction. pSPMs: pNENs patients with second primary malignancies.

The established nomogram included all significant independent prognostic factors identified in the cox analysis (Figure 6(a)). According to the nomogram, the latency period showed the largest range of scores, indicating that it had the greatest impact on the OS. The C-index of the nomogram was 0.765, and the adjusted value was 0.755 after bootstrapping. Calibration plots demonstrated that 5-, 10-, and 15-year OS rates were close to the actual OS rates, proving good calibration of the nomogram (Figure 6(b)). As shown in Figure 6(c), the AUC of the nomogram for the 5-, 10-, and 15-year OS were 84.42, 78.16, and 70.12%, respectively, which were superior to other variables alone. Moreover, the results of DCA showed that the nomogram has a high net benefit and net reduction. These results suggested that the nomogram performed well in the prediction of OS in pSPMs (Figure 6(d)).

## 4. Discussion

In this study, we revealed the risk factors for developing SPMs in patients with pNENs and investigated independent prognostic factors in pSPMs. Nicholas et al. found that the most common SPM site is the lung in patients with cancer [24]. Another study showed a systematic connection between the SPM position and the primary tumor position; patients with primary gynecologic tumors are also prone to develop SPMs in the female genital system or female breast [25]. However, we found that the most common SPM site was the prostate gland, followed by the female breast and the intestinal tract, which was inconsistent with previous results. This finding will be valuable for surveillance strategies in patients with pNENs.

In terms of the risk factors for developing SPMs after pNENs, our study confirmed that treatment without surgery and diagnosed in recent periods were significantly associated with an increased risk of developing SPMs. Jia et al. reported that treatment with surgery was associated with an increased risk of developing SPMs and a good prognosis in patients with colorectal cancer who more likely to develop SPMs because of a longer expected lifespan [26]. Chen et al. also reported patients with esophageal cancer who received surgery had an increased risk of developing SPMs [27]. However, we found that treatment with surgery was associated with a decreased risk of developing SPMs, which was inconsistent with previous conclusions. We thought this discrepancy might be attributed to tumor heterogeneity. A prior study showed that diagnosis in recent periods was significantly associated with an increased risk of developing SPMs after lung neuroendocrine tumors [28]. This result was consistent with our findings. The increased occurrence of SPM in recent periods might be due to medical advances that led to a longer expected lifespan and patient's increased health consciousness that resulted in more regular return visit after first tumor diagnosis.

Although a few studies have reported the factors associated with the prognosis of pNENs, studies on the prognostic factors for the development of pSPMs are limited. Our results clarified that age, SEER stage, latency period, surgery, and radiotherapy are independent prognostic factors in pSPMs. Several studies have reported that treatment with radiotherapy or surgery was independently correlated with prognosis of cancer patients with SPMs [29–32]. We showed that radiotherapy or surgery plays a favorable role in the prognosis of pSPMs. In the current study, we confirmed that the SEER stage is an independent prognostic indicator, which is in agreement with the findings of Yang et al. [31]. In terms of age at diagnosis, former studies have revealed that older age was a significant risk factor for OS in cancer survivors with SPMs [32, 33], and we found a similar result in this study. An increasing number of studies have reported that a longer latency period was associated with a better OS [34–36]. Our study also revealed that a longer latency period correlated with better OS in pSPMs.

This study has several limitations. First, since this was a retrospective study, selection bias could not be avoided [37]. Second, because of the nature of the SEER database, we could not obtain information on the effect of certain parameters, such as detailed treatment information, family history, and history of smoking and drinking, on the risk of developing SPMs in patients with pNENs and the prognosis of pSPMs. Finally, our nomogram model still needs further external validation. Our findings will help improve strategies for the surveillance of risk factors associated with the development of SPMs in patients with pNENs and prognostic risk factors in pSPMs.

## 5. Conclusions

In summary, patients with pNENs who did not undergo surgery and who were diagnosed at a later period had higher risks of developing SPMs. For pSPMs, we successfully created a nomogram to predict 5-, 10-, and 15-year OS based on independent prognostic predictors. Our findings have clinical implications for the prevention and surveillance of SPM occurrence among pNEN survivors and improvement of prognosis among pSPMs.

## Figures and Tables

**Table 1:** Demographic and clinical characteristics of pancreatic neuroendocrine neoplasms patients with second primary malignancies.

Variables	Count (%)
*Gender*	
Female	146 (44.79)
Male	180 (55.21)
*Age*	
18–44 years	46 (14.11)
45–64 years	151 (46.32)
≥65 years	129 (39.57)
*Marital status*	
Married	236 (72.39)
Unmarried	74 (22.70)
Unknown	16 (4.91)
*Primary site*	
Head	87 (26.69)
Body	49 (15.02)
Tail	106 (32.52)
Others	84 (25.77)
*Chemotherapy*	
Yes	47 (14.42)
No/unknown	279 (85.58)
*Radiotherapy*	
Yes	13 (3.99)
No/unknown	313 (96.01)
*Surgery*	
Yes	237 (72.70)
No/unknown	89 (27.30)
*SEER stage*	
Localized	134 (41.10)
Regional	76 (23.31)
Distant	101 (30.98)
Unknown	15 (4.61)
*Tumor function*	
Yes	92 (28.22)
No	234 (71.78)
*Race*	
White	268 (82.21)
Black	32 (9.82)
Others	26 (7.97)
*Year of diagnosis*	
1988–1997	36 (11.04)
1998–2007	121 (37.12)
2008–2016	169 (51.84)
*Tumor size*	
<2 cm	72 (22.09)
2–4 cm	98 (30.06)
>4 cm	115 (35.28)
Unknown	41 (12.57)
*Latency period*	
≤12 months	76 (23.31)
13–60 months	141 (43.25)
61–120 months	77 (23.62)
121–180 months	23 (7.06)
≥181 months	9 (2.76)
*Tumor type*	
NET	324 (99.39)
NEC	2 (0.61)
*Tumor grade*	
1	139 (42.64)
2	36 (11.04)
3 + 4	11 (3.38)
Unknown	140 (42.94)

**Table 2:** Baseline characteristics of unmatched and matched cohorts.

Characteristics	Unmatched cohort			Matched cohort		
	No SPMs (*n* = 3333)	SPMs (*n* = 173)	*p* value	No SPMs (*n* = 865)	SPMs (*n* = 173)	*p* value
*Sex*			0.997			0.759
Male	1773 (53.2)	92 (53.1)		471 (54.5)	92 (53.1)	
Female	1560 (46.8)	81 (46.9)		394 (45.5)	81 (46.9)	
*Race*			0.483			0.824
White	2607 (78.2)	142 (82.1)		709 (81.9)	142 (82.1)	
Black	393 (11.8)	17 (9.8)		76 (8.9)	17 (9.8)	
Others	333 (10.0)	14 (8.1)		80 (9.2)	14 (8.1)	
*Age at initial diagnosis*			0.252			0.890
18–44 years	580 (17.4)	26 (15.6)		118 (13.6)	26 (15.6)	
45–64 years	1662 (49.9)	80 (46.2)		407 (47.1)	80 (46.2)	
≥65 years	1091 (32.7)	67 (38.2)		340 (39.3)	67 (38.2)	
*Marital status*			0.018			0.731
Yes	2173 (65.2)	128 (73.9)		629 (72.7)	128 (73.9)	
No	1160 (34.8)	45 (26.1)		236 (27.3)	45 (26.1)	
*Year of initial diagnosis*			<0.001			0.230
1988–1997	83 (2.5)	5 (2.9)		47 (5.4)	5 (2.9)	
1998–2007	517 (15.5)	51 (29.5)		218 (25.2)	51 (29.5)	
2008–2016	2733 (82.0)	117 (67.6)		600 (69.4)	117 (67.6)	
*Primary site*			0.043			0.472
Head	1049 (31.5)	37 (21.4)		234 (27.1)	37 (21.4)	
Body	477 (14.3)	26 15.0)		115 (13.3)	26 (15.0)	
Tail	1198 (35.5)	74 (42.8)		341 (39.4)	74 (42.8)	
Others	609 (18.7)	36 (20.8)		175 (20.2)	36 (20.8)	
*Type*			0.118			0.901
NET	3223 (96.7)	171 (98.8)		854 (98.7)	171 (98.8)	
NEC	110 (3.3)	2 (1.2)		11 (1.3)	2 (1.2)	
*Functional*			<0.001			0.973
Yes	356 (10.7)	37 (21.4)		186 (21.5)	37 (21.4)	
No	2977 (89.3)	136 (78.6)		679 (78.5)	136 (78.6)	
*Tumor size*			0.292			0.454
<2 cm	817 (24.6)	48 (27.7)		217 (25.1)	48 (27.7)	
2–4 cm	1273 (38.1)	56 (32.4)		323 (37.3)	56 (32.4)	
>4 cm	1243 (37.3)	69 (39.9)		325 (37.6)	69 (39.9)	
*Surgery*			<0.001			0.964
Yes	2618 (78.5)	155 (95.1)		776 (89.7)	155 (95.1)	
No/unknown	715 (21.5)	18 (4.9)		89 (10.3)	18 (4.9)	
*Radiotherapy*			0.166			0.600
Yes	176 (5.3)	5 (2.9)		32 (3.7)	5 (2.9)	
No/unknown	3157 (94.7)	168 (97.1)		833 (96.3)	168 (97.1)	
*Chemotherapy*			0.005			0.757
Yes	634 (19.1)	18 (10.4)		97 (11.2)	18 (10.4)	
No/unknown	2699 (80.9)	155 (89.6)		768 (88.8)	155 (89.6)	
*Grade*			0.003			0.743
1	2145 (64.4)	129 (74.6)		652 (75.4)	129 (74.6)	
2	676 (20.3)	33 (19.1)		148 (17.1)	33 (19.1)	
3 + 4	512 (15.3)	11 (6.3)		65 (7.5)	11 (6.3)	
*SEER stage*			0.006			0.462
Localized	1452 (43.6)	93 (53.8)		421 (48.7)	93 (53.8)	
Regional	797 (23.9)	43 (24.9)		245 (28.3)	43 (24.9)	
Distant	1084 (32.5)	37 (21.3)		199 (23.0)	37 (21.3)	

Abbreviations: SPMs, second primary malignancies.

## Data Availability

Publicly available datasets were analyzed in this study. The data used and/or analyzed in this study are available in the Surveillance, Epidemiology, and End Results (SEER) Database of the National Cancer Institute (http://seer.cancer.gov).
